# Gradual Optimization of Headspace Solid-Phase Microextraction Conditions of Volatiles in Pepper Chicken Soup Combined with Gas Chromatography-Mass Spectrometry and Principal Component Analysis

**DOI:** 10.1155/2019/8963191

**Published:** 2019-04-01

**Authors:** Qiuyue Geng, Ping Zhan, Honglei Tian, Peng Wang, Haitao Chen

**Affiliations:** ^1^College of Food Engineering and Nutritional Science, Shaanxi Normal University, Xi'an, 710119, China; ^2^Food College of Shihezi University, Shihezi 832000, China; ^3^School of Food and Chemical Engineering of Beijing Technology and Business University, Beijing 102488, China

## Abstract

A single-factor gradual optimization method was developed in this experiment in order to improve the headspace solid-phase microextraction (HS-SPME) effect of volatile compounds in pepper chicken soup. The different extraction conditions included fibers with different coating materials, sample volume, extraction temperature, and extraction time. The total peak areas and the numbers of valid peaks were compared and analyzed as the indicators of condition optimization. Gas chromatography-mass spectrometry (GC-MS) results showed that the four factors all have significant impact on the extraction effect of volatiles in pepper chicken soup. Using the principal component analysis (PCA), the optimal conditions of HS-SPME were inferred below: an extraction fiber of 50/30*μ*m DVB/CAR/PDMS, a sample volume of 7 g, an extraction temperature of 65°C, and an extraction time of 30 min. Compared to the original extraction conditions, the optimized conditions were especially advantageous for the comprehensive analysis of volatiles, which could be potentially used in further study of soup.

## 1. Introduction

Pepper chicken is a traditional and characteristic food in Xinjiang, China; in addition to its rich nutrition, the delicious flavor of its soup has won great popularity among the local and even national consumers in recent years. Spices, including chili pepper (*Capsicum annuum L*.), pricklyash peel (*Zanthoxylum bungeanum M*.), and scallion, can be properly deep-fried or stewed to be prepared for different seasoning oils and sauces, which combined with the original stewed chicken soup can be blended into exquisite flavor soup of pepper chicken. However, flavor sensations are highly influenced by the quantitative and qualitative combination of aroma compounds in food [1]. Accordingly, an efficient extraction of volatiles in pepper chicken soup is an important prerequisite for identifying its aroma substances. How to analyze the flavor substances scientifically and accurately is the key problem to evaluate the flavor quality of products [2].

Headspace solid-phase microextraction (HS-SPME) technique is a widely used pretreatment method for flavor detection [3–5]. Compared with traditional extraction methods such as distillation extraction and organic solvent extraction, HS-SPME is more convenient and fast in preparing test samples. And it can recognize more volatile organic compounds (VOCs) than headspace extraction method. In particular, HS-SPME has some obvious advantages, such as zero sample loss and zero pollution, as well as favorable sensitivity. So far, it has been applied to the analysis of volatile and semivolatile substances [6] in beef [7], duck [8], strawberry [9], and other foods. In order to improve the extraction efficiency of SPME, it is an indispensable step to optimize the extraction conditions. The effects of extraction are affected by various external conditions, among which the type of fiber coating, sample volume, extraction temperature, and time are representative [10, 11]. After optimizing these conditions, different researchers got various analytical results of soy sauce, pepper, roasted lamb [12–14], and so on. Gas chromatography-mass spectrometry (GC-MS) is a commonly used detection method, which can display the content of each volatile substance in different peak areas, and we can learn the distribution and proportion of different kinds of substances [15]. Thus changes of peak areas and numbers often reflect changes of types and contents of volatile compounds [16, 17]. Predecessors had proved the validity of using the number of peaks and total area of chromatogram as criteria of extraction performance in SPME optimization studies [13, 18]. Multivariate statistical tools like response surface methodologies are usually used in optimization experiment, which has not only advantages but also drawbacks [19]. The single-factor gradual optimization method has been used by few people. However, its experiment amount is lower than orthogonal design under four factors, five levels (L_25_ (5^6^)) [20]. By contrast, the single-factor gradual optimization method is more suitable for this study. In this study, the HS-SPME conditions of volatiles in pepper chicken soup were optimized to obtain the best extraction conditions and effect by means of single-factor gradual optimization, and provide necessary conditions for the identification of aroma and assistance for further processing study of pepper chicken soup.

## 2. Experimental

### 2.1. Materials and Pretreatment

Pepper chicken soup was from Gazi Pepper Chicken Restaurant, Shihezi City, Xinjiang, China. The restaurant was selected using a questionnaire survey. Firstly, statistics were collected on eating houses with pepper chicken in Shihezi area, and then the flavor and taste of the dish were evaluated by designing questions. The questionnaires were distributed in the bustling area of the city, and restaurants with more than 90% favorable rates were selected as alternative sample sources. Finally it was determined to take soup sample from Gazi Pepper Chicken Restaurant by drawing lots. Before analysis, the soup sample was stored in a refrigerator at −20°C. The standard solution of n-alkane (C_8_~C_20_) was purchased from Sigma-Aldrich (St. Louis, MO, USA) and stored at 4°C in a fridge.

### 2.2. Optimization of SPME

The four conditions which influence extraction effect significantly were determined by early experimental work, containing the type of fibers, sample volume, extraction temperature, and extraction time. Once the best fiber is selected, the other SPME conditions can be optimized in the order above. By combining a great deal of research on SPME conditions, the original sample volume was set as 5 g, the original extraction temperature was set as 60°C, and the original extraction time was set as 30 min.

#### 2.2.1. Optimization of Fibers

The first step was the choice of extraction fiber. Five different coating fibers purchased from Supelco (Inc., Bellefonte, PA), namely, 85*μ*m CAR/PDMS, 65*μ*m PDMS/DVB, 100*μ*m PDMS, 50/30*μ*m DVB/CAR/PDMS, and 85*μ*m PA, were prepared and conditioned at the GC injection port in accordance with operating instructions as in Table 1.

After pre-experiment, the sample volume was set as 5 g (placed into the 15 mL vial), the extraction temperature 60°C (regulated by a digital display constant temperature water bath), and the extraction time 30 min. The five fibers were used to extract volatile compounds in pepper chicken soup, and samples extracted with different conditions were analyzed by GC-MS, respectively. By comparing the total peak area and the effective number of peaks, the results of extracting volatiles in pepper chicken soup were investigated.

#### 2.2.2. Optimization of Sample Volume

After shaking the soup sample to make the oil phase and water mixed evenly, 4 g, 5 g, 6 g, 7 g, and 8 g of shaken soup were separately packed into five identical headspace vials. Then volatile components were extracted by the best fiber selected in the previous step. Finally the impact of sample volume on peak area and quantity was inspected.

#### 2.2.3. Optimization of Extraction Temperature

Since the optimal fiber and sample volume were selected, the same volume of samples was absorbed separately at temperatures of 50°C, 60°C, 65°C, 70°C, and 80°C. Other conditions were kept unchanged, and the extraction effect of volatile substances in pepper chicken soup was explored.

#### 2.2.4. Optimization of Extraction Time

The extraction fiber type, sample volume, and extraction temperature were set as constant parameters, and the durations were set at 20 min, 30 min, 40 min, 50 min, and 60 min. The optimal extraction time would be set with other invariant conditions.

#### 2.2.5. GC-MS Analysis

The analysis of volatile compounds was conducted by 7890A-5977B GC-MS detection system (Agilent Technologies, USA). The fused silica capillary column HP-INNOWax (30 m×0.25 mm ID, 0.25 *μ*m film thickness) was used to perform the chromatographic separation of volatiles. The oven temperature was programmed as follows: the initial temperature of the column was held at 40°C for 3 min, then increased to 200°C at the speed of 5°C/min, maintained for 5 min, lastly increased to 230°C at the speed of 10°C/min, and remained for 5 min. The injector temperature was 230°C and helium was used as the carrier gas with a flow rate of 1.0 mL/min and splitting mode 20:1. Mass spectra parameters covered electron impact ionization with electron energy of 70 eV, ion source temperature 230°C, MS quad temperature 150°C, and mass scan range m/z 30~750.

Identifications of unknown compounds were realized by matching GC-MS data and databases (NIST 14 and Wiley 10), selecting the components with similarity greater than 60 (maximum 100) and combining them with the retention indices.

### 2.3. Statistical Analysis

Data statistics and mapping analysis were carried out by SPSS 23.0 and Origin 9.0. All experiments were run three times in parallel and the data were expressed as means ± standard errors. Analysis of variance (ANOVA) and Duncan's multiple range tests were applied to evaluate the significance of data sets. Differences were considered statistically significant (*p*<0.05).

## 3. Results and Discussion

### 3.1. Fiber Selection

**Figure 1:**
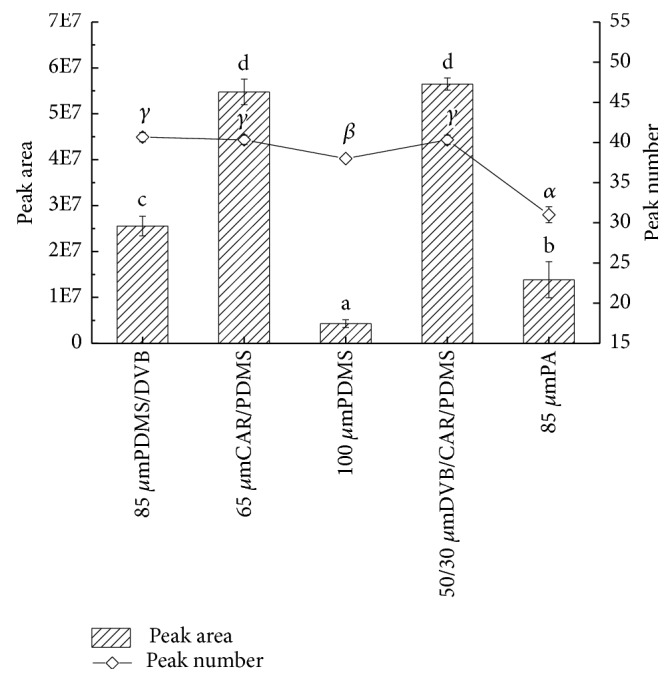
Effects of different fibers on extracting volatiles of pepper chicken soup. English letters “a”, “b”, “c”, “d” indicate Duncan's multiple range test among peak areas of the five fibers. Greek letters “*α*”, “*β*”, “*γ*” indicate Duncan's multiple range test among peak numbers of the five fibers. Data in the same group marked with different letters mean significant difference (*p*<0.05) and the same letters denote not significant difference (*p*<0.05).

The polarity of fibers is determined by different coating materials [21], which allow fibers to reflect different peak areas. The peak areas and effective numbers of peaks of the five fibers were compared, as results show in Figure 1. PDMS/DVB fiber adsorbed the most species of volatile compounds from pepper chicken soup, closing to fibers CAR/PDMS and DVB/CAR/PDMS, while PA fiber showed the weakest adsorption effect. In addition, total peak areas acquired from fibers PDMS/DVB, PDMS, and PA were significantly lower than areas of fibers CAR/PDMS and DVB/CAR/PDMS. Total peak area of volatile substances extracted by CAR/PDMS (5.5 × 10^7^) was highly consistent with area by DVB/CAR/PDMS fiber (5.6 × 10^7^). Meanwhile, the amount of volatile compounds obtained from CAR/PDMS equaled the amount from DVB/CAR/PDMS fiber. In conclusion, both of CAR/PDMS and DVB/CAR/PDMS fibers showed fine extraction ability of volatiles in pepper chicken soup. Alternatively, it was necessary to further compare the extraction results of each sort of volatiles in pepper chicken soup.

**Figure 2:**
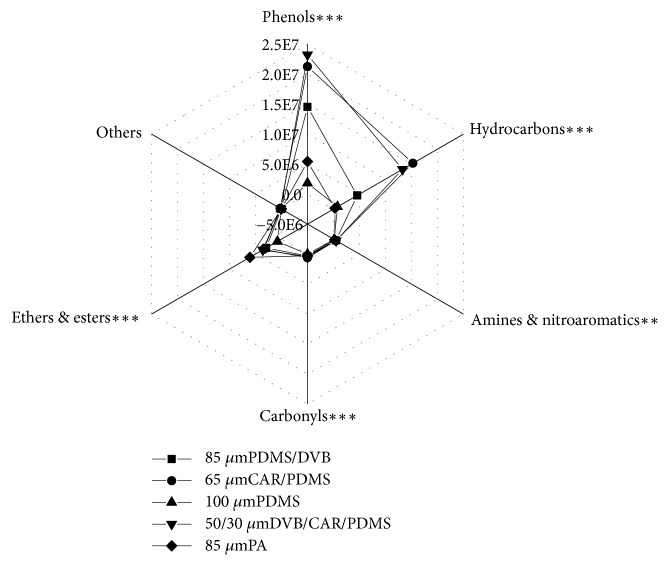
Radar plot of extraction effect on extracting six components by the five fibers. Compounds which are assessed in six categories are marked with “*∗*” to dedicate significant level in the same group. *∗∗* Difference is significant (p<0.01); *∗∗∗* Difference is extremely significant (p<0.001).

Differences in polarity caused by coating materials bring force diversity of SPME fibers ability to extract various kinds of volatile components [11]. Based on previous mass researches, adsorption properties of different fibers are summarized below. Fiber PDMS/DVB is appropriate for detection of trace volatile flavor compounds and can be used for extracting semivolatile polar substances and amines, monitoring volatiles in pesticides well [22]. Fiber PDMS is very friendly to extracting micromolecular nonpolar volatiles [13, 22]. Fiber PA works well when its action objects are polar semivolatiles and phenols [13, 22]. DVB/CAR/PDMS and CAR/PDMS are bipolar coating types, which are considered to be more efficient [22], while it is proved that fiber DVB/CAR/PDMS is widely used in aroma analysis of meat products [23, 24], which can extract extensive substances from C_3_ to C_20_. Six kinds of volatile components of pepper chicken soup were absorbed by five kinds of fibers (Figure 2). As seen in the picture, extraction results of amines, nitroaromatics, carbonyls, and others (including sulfur compounds and heterocycles) were almost unanimous, but results of phenols, hydrocarbons, ether, and esters were distinguished well. Fiber DVB/CAR/PDMS showed strong extraction ability for phenols; fiber CAR/PDMS displayed excellent capacity to adsorb hydrocarbons; fiber PDMS/DVB performed ordinarily on the whole; ether and esters were collected favorably as exhibited by fiber PA, but phenols and hydrocarbons were collected badly; outcome of extraction experiment by fiber PDMS was the worst, showing prominent impact on no sort of component. Judged synthetically, fibers DVB/CAR/PDMS and CAR/PDMS with good sensitivity still remained the best suitable fibers among the others when served for extracting volatiles in pepper chicken soup.

**Figure 3:**
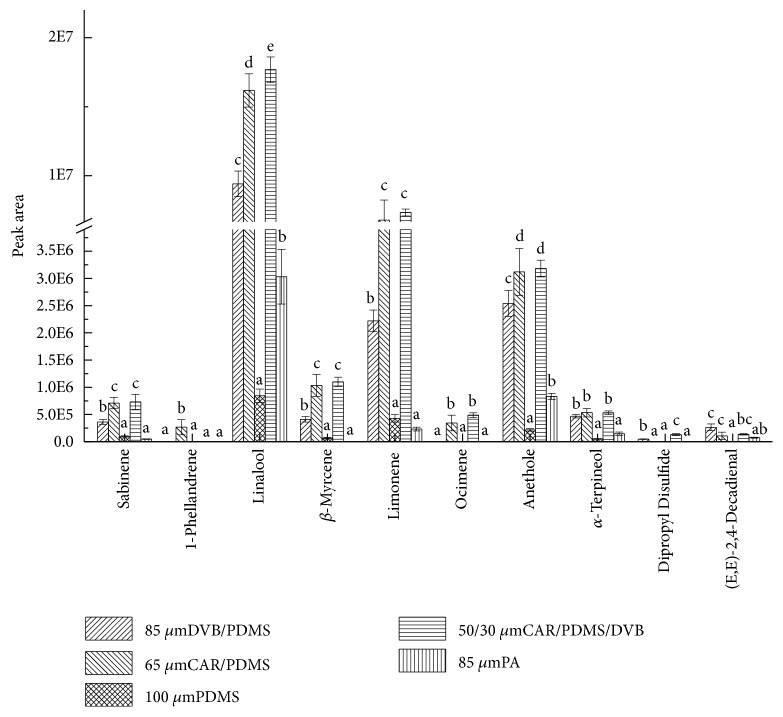
Effects of the five fibers on peak areas of the ten key aroma compounds. English letters “a”, “b”, “c”, “d”, “e” indicate Duncan's multiple range test among peak areas of the five fibers. Data in the same compound marked with different letters mean significant difference (*p*<0.05) and the same letters denote not significant difference (p<0.05).

According to an existing research [25], sabinene, *β*-myrcene, and linalool were the key aroma compounds in pepper chicken soup, along with (E, E)-2,4-decadienal, ocimene, and so on. The ten pivotal aroma compounds are compared in terms of peak area determination, including sabinene, 1-phellandrene, linalool, myrcene, limonene, ocimene, anethole, *α*-terpineol, dipropyl disulfide, and (E,E)-2,4-decadienal, as shown in Figure 3. Obviously, the figure sheds light on scattered differences of these valid compounds caused by the five types of fibers. Peak areas of linalool, limonene, and anethole obtained from fibers CAR/PDMS and DCB/CAR/PDMS were much higher than others. Among the ten compounds, seven were partial to DVB/CAR/PDMS fiber by means of placing their biggest areas in DVB/CAR/PDMS, including linalool, limonene, ocimene, myrcene, sabinene, anethole, and dipropyl disulfide. Peak area of (E, E)-2,4-decadienal from DVB/CAR/PDMS was higher than from CAR/PDMS, but lower than from PDMS/DVB. Dipropyl disulfide was found in analytes collected by both PDMS/DVB and DVB/CAR/PDMS fibers, while it was undetected by CAR/PDMS, PDMS, and PA. Areas of *α*-terpineol were similar to each other referring to PDMS/DVB, CAR/PDMS, and DVB/CAR/PDMS fibers, yet areas were apparently lower than PDMS and PA fibers. Considering all aspects, DVB/CAR/PDMS with three-layer composite coating materials was the best in slot fiber for extracting either total volatile components or key compounds in pepper chicken soup.

### 3.2. Confirmation of Sample Volume

**Figure 4:**
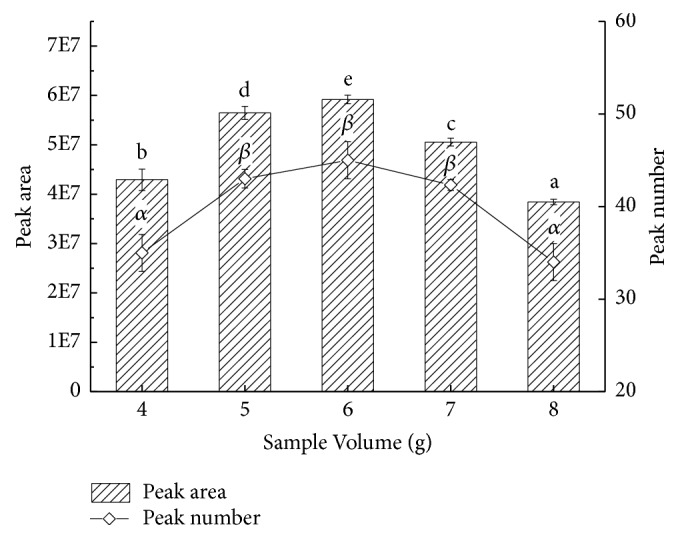
Effects of different sample volumes on extracting volatiles of pepper chicken soup. English letters “a”, “b”, “c”, “d”, “e” indicate Duncan's multiple range test among peak areas of the five sample volumes. Greek letters “*α*” and “*β*” indicate Duncan's multiple range test among peak numbers of the five sample volumes. Data in the same group marked with different letters mean significant difference (*p*<0.05) and the same letters denote not significant difference (p<0.05).

As the fiber DVB/CAR/PDMS was selected, 4 g, 5 g, 6 g, 7 g, and 8 g pepper chicken soup were measured out separately, and the effect of sample volume on extracting volatiles in pepper chicken soup were explored (Figure 4). Extraction temperature and time remained ditto. In the diagram, the change trend of peak area and quantity was evident, both of which were increasing firstly and then deceasing. When sample volume reached 6 g, peak area and quantity reached their acmes at the same time. Sample size presented duality, which was due to the interaction between sample volume and headspace volume. With a too small size of sample volume, available volatile substances were too little; thus the extraction effect was affected. From 4 to 6 g, with the increasing of sample volume, volatile components increased as well. From 6 to 8 g, with the increasing of sample liquid volume in the headspace bottle, the pressure inside the liquid became larger, and the headspace became smaller, which limited the volatilization of the aroma components, preventing them from evaporating from the soup; thus the extraction result of SPME deteriorated in this case. Judged preliminarily, the feasible sample volume was 5 to 7 g.

**Figure 5:**
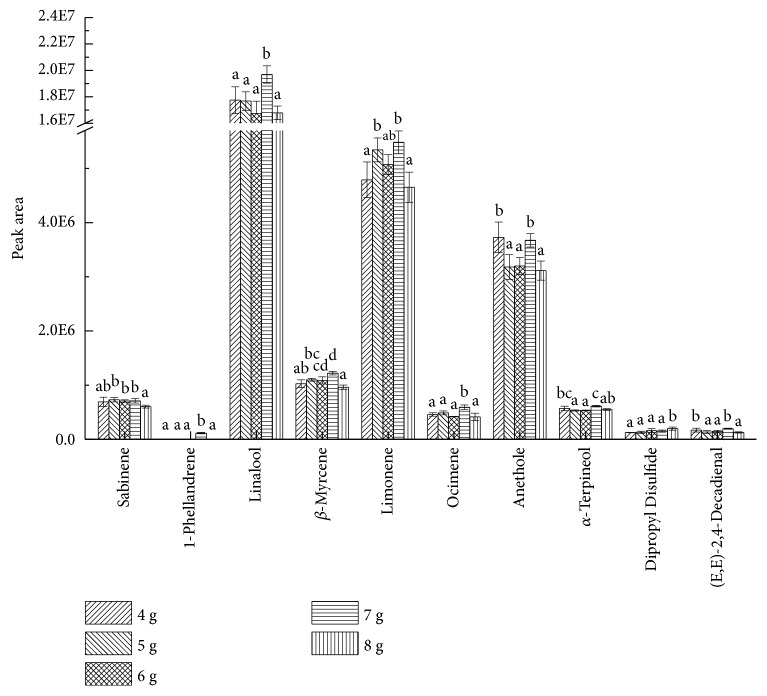
Effects of different sample volume on peak areas of key aroma compounds. English letters “a”, “b”, “c”, “d” indicate Duncan's multiple range test among peak areas of the five sample volumes. Data in the same compound marked with different letters mean significant difference (*p*<0.05) and the same letters denote not significant difference (p<0.05).

To verify the optimal sample volume of SPME of pepper chicken soup, peak areas and quantities of ten major compounds, including sabinene and linalool, are contrasted, as shown in Figure 5. It can be seen that the peak areas of linalool, *β*-myrcene, limonene, ocimene, *α*-terpineol, and (E, E)-2,4-decadienal reached their acmes at 7 g sample volume. In that case, peak area of anethole reached the second-highest value, whose column was only lower than column at 4 g sample volume, while the variation of areas from sabinene and dipropyl disulfide was tiny. According to what discussed above, the best sample volume was 7 g, agreeing with the conclusion based on referring to total area and number.

### 3.3. Effect of Extraction Temperature

**Figure 6:**
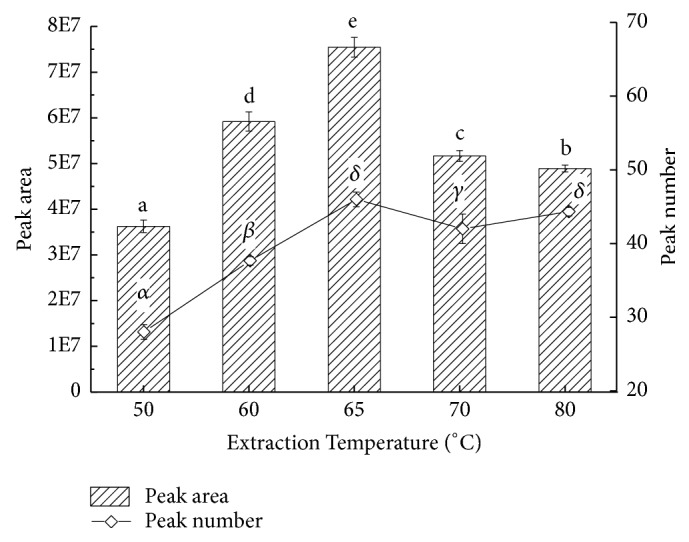
Effects of extraction temperature on extracting volatiles of pepper chicken soup. English letters “a”, “b”, “c”, “d”, “e” indicate Duncan's multiple range test among peak areas of the five extraction temperatures. Greek letters “*α*”, “*β*”, “*γ*”, “*δ*” indicate Duncan's multiple range test among peak numbers of the five extraction temperatures. Data in the same group marked with different letters mean significant difference (*p*<0.05) and the same letters denote not significant difference (*p*<0.05).

Temperature is one of the most important factors affecting the performance of SPME. Using the selected fiber 50/30 *μ*m DVB/CAR/PDMS, measuring 7 g of soup sample, extraction experiment was carried out at temperatures of 50°C, 60°C, 65°C, 70°C, and 80°C. Extraction process lasted for 30 min and then collections were desorbed by GC-MS apparatus, in which case influence of temperature on the course of extraction was inspected (Figure 6). Columns of total peak areas presented earlier increase and later decrease trend in the figure. When extraction temperature was between 50°C and 65°C, the peak area and number both increased; when extraction temperature was between 65°C and 80°C, changes in the peak numbers tended to be gentle with the decreasing of the peak areas. This was due to the fact that the effect of temperature is double. On the one hand, before the optimal temperature, the motion of molecules increased with temperature increasing, which benefited volatile compounds to be spared from the soup and then the concentration of substances in the headspace was fortified, so that absorbing became easier for the fiber. On the other hand, the adsorption capacity of fiber would be reduced at an over-rising temperature, causing the descending of adsorption amount [26]. Peak area and number both reached the maximums at the same time when the temperature was 65°C. Judged reasonably, the best extraction temperature was 65°C.

**Figure 7:**
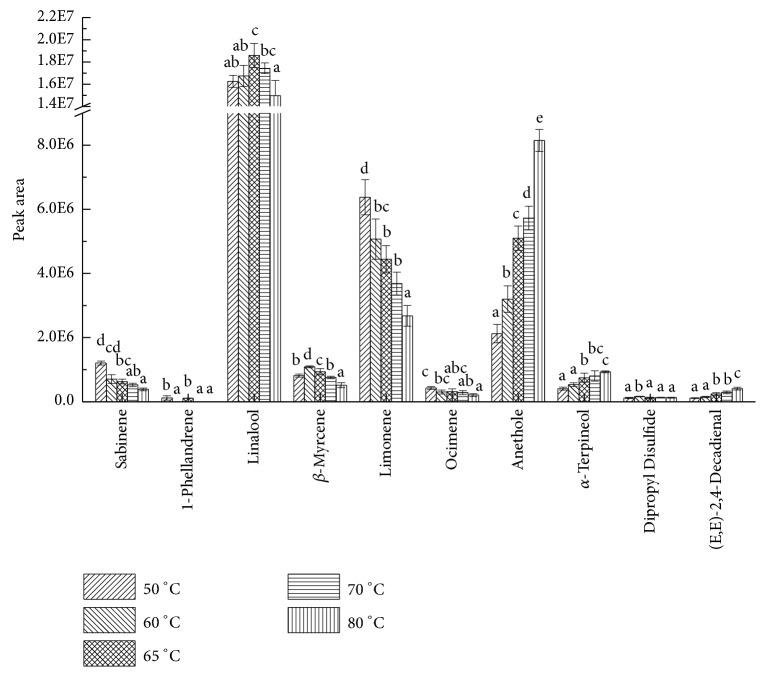
Effects of extraction temperature on peak areas of key aroma compounds. English letters “a”, “b”, “c”, “d”, “e” indicate Duncan's multiple range test among peak areas of the five extraction temperatures. Data in the same compound marked with different letters mean significant difference (*p*<0.05) and the same letters denote not significant difference (p<0.05).

Comparison of peak areas of key volatiles at different extraction temperatures is shown in Figure 7. As seen from it, extraction was affected by temperature diversely, and the variation trend of each component was obviously different. Effects of temperature on linalool and *β*-myrcene showed visible two-sidedness, first increasing and then decreasing. The peak area of linalool reached its peak at 65°C, and the peak area of *β*-myrcene at 60°C. The peak areas of anethole, *α*-terpineol, and (E, E)-2,4-decadienal showed an uptrend with the increasing of extraction temperature. Dissecting the cause, appropriate heating accelerated the motion of molecules in the soup sample; thus it increased the concentration of analytes in the headspace, thereby improving the extraction results and analytical sensitivity. The extraction and adsorption were exothermic processes, so an over-temperature would cause an over-saturated situation of the headspace steam, decline of distribution coefficient, and finally reduction of absorbability. Moreover, peak areas of linalool, sabinene, and ocimene descended with the increase of extraction temperature, which were inferred to be consequence of chemical reactions at an over-temperature. It has been reported that limonene could be oxidized to carvone [27] or dehydrogenated to para-cymene [28] under certain conditions. It can also generate *α*-terpineol [29] and terpin hydrates with water molecules in the presence of mineral acids. Area of dipropyl disulfide showed no significant change; in addition, 1-phellandrene was only detected at 65°C and 40°C. Considering the instability of some aroma active substances in pepper chicken soup, they may be decomposed or converted to other compounds due to sizzling temperature [30]; thus in summary, the best extraction temperature was 65°C.

### 3.4. Effect of Extraction Time

**Figure 8:**
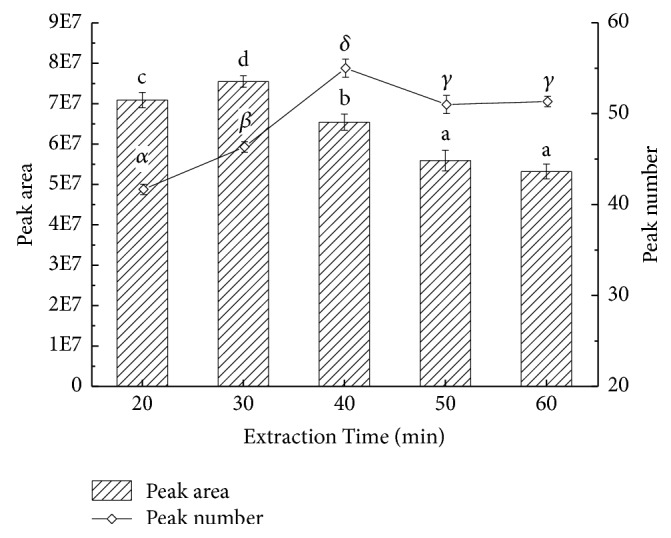
Effects of extraction time on volatile compounds of pepper chicken soup. English letters “a”, “b”, “c” indicate Duncan's multiple range test among peak areas of the five extraction times. Greek letters “*α*”, “*β*”, “*γ*”, “*δ*” indicate Duncan's multiple range test among peak numbers of the five extraction times. Data in the same group marked with different letters mean significant difference (*p*<0.05) and the same letters denote not significant difference (*p*<0.05).

Under the optimal conditions including fiber type, sample volume, and extraction temperature, how extraction time played role in extracting volatile compounds from pepper chicken soup was investigated by setting gradient duration as 20 min, 30 min, 40 min, 50 min, and 60 min (Figure 8). As seen, peak area and number both firstly increased and then decreased as well as tending to be gentle. With a short time of extraction, parts of volatiles in the headspace were too late to enter the fiber, which impeded the fiber to adsorb enough analytes, whereupon the peak area occurred small; with a too long extraction time, chemicals' extraction equilibrium had been reached, but due to the competitive adsorption, some compounds were lost from the fiber [31], with a declining peak area finally. The peak area and number reached their peaks at 30 min and 40 min, respectively, inferring that the desired duration of extraction was between 30 min and 40 min.

**Figure 9:**
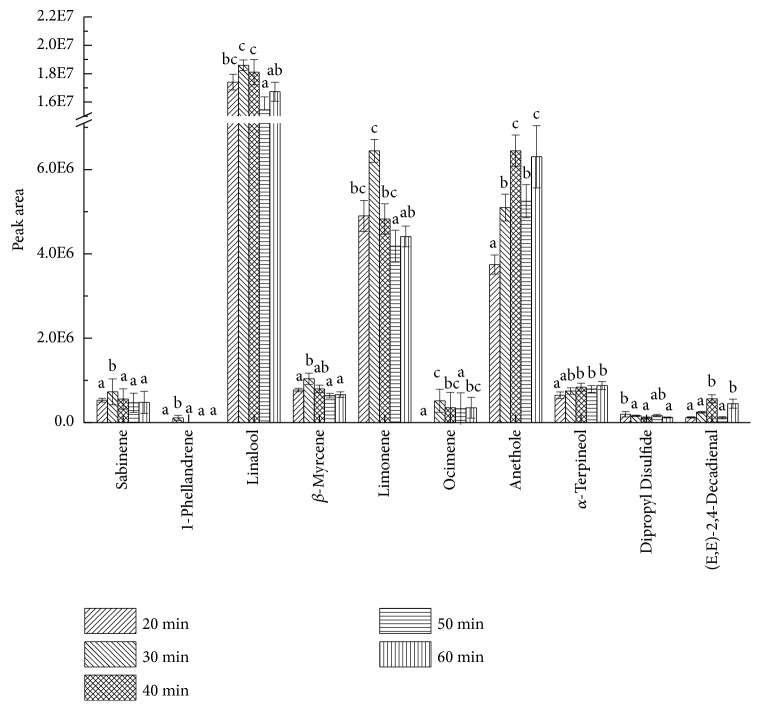
Effects of extraction time on peak areas of key volatile compounds. English letters “a”, “b”, “c” indicate Duncan's multiple range test among peak areas of the five extraction times. Data in the same compound marked with different letters mean significant difference (*p*<0.05) and the same letters denote not significant difference (p<0.05).

Although extraction time had the tendency to regulate total area overall, the impacts on individual components were various. Concerning properties of volatiles themselves, there was no uniform trend in the adsorption of different substances by the fiber. The effect of time on extracting the peak area of dominant sectors is shown in Figure 9. It could be seen that more than half of the ten key compounds reached their largest values of peak area at 30 min, including sabinene, linalool, myrcene, limonene, ocimene, and 1-phellandrene. Moreover, 1-phellandrene was detected at 30 min only. Two compounds, anethole and (E, E)-2,4-decadienal, reached their peak values at 40 min. Peak area of *α*-terpineol increased slowly. Judged comprehensively, extraction was better at 30 min.

**Figure 10:**
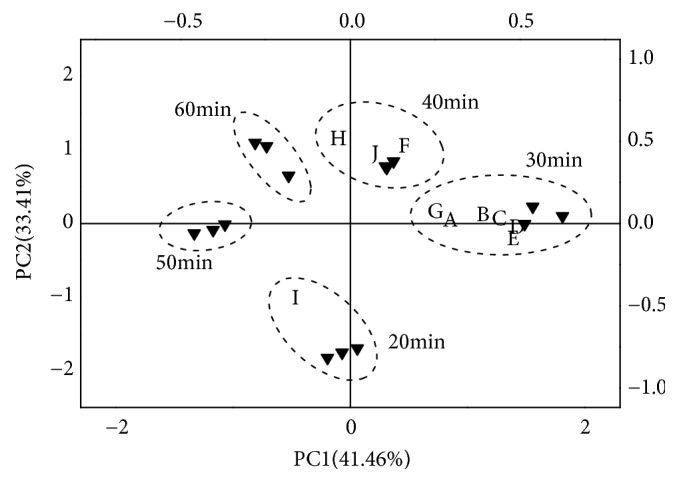


By principal component analysis (PCA), multidimensional and complex data can be simplified, the characteristic information of samples can be extracted, so as to evaluate differences and similarities among samples clearly [15]. Results of PCA are usually marked with two percentages, the first principal component (PC1) and the second principal component (PC2). PCA of the peak area data of key aroma substances under different extraction time are shown in Figure 10. PC1 was 41.46% and PC2 33.41%. The cumulative contribution attained 74.87%, which could represent the sample information preferably. It can be seen from the picture that three parallel treatments with different temperatures were located in different areas of the loading plot and gathered together respectively, indicating good repeatability of the test. It also illustrated that satisfactory distinction could be made among the soup samples by PCA analysis. In the PCA figure, sabinene, 1-phellandrene, linalool, myrcene, limonene and anethole were surrounding treatments of 30 min, explaining there was some correlation between these compounds and the flavor of pepper chicken soup at 30 min. The third group of samples were surrounded by ocimene, *α*-terpineol and (E, E)-2,4-decadienal, which contributed more to the flavor formation of soup samples under 40 min treatment. Dipropyl disulfide had changed greatly when extraction time was set as 20 min. To sum up, the best extraction was at 30 min.

### 3.5. Optimization Effect Analysis

The optimal extraction conditions were used to analyze volatile compounds in pepper chicken soup, and results before and after optimization were compared as shown in Table 2. The extraction effect was chosen using fiber 50/30 *μ*m DVB/CAR/PDMS in the first step with sample volume 5 g, extraction time 30 min, and extraction temperature 60°C as the original effect. And the final extraction effect resulted from optimal conditions with fiber DVB/CAR/PDMS, sample volume 7 g, extraction temperature 65°C, and extraction time 30 min. In Table 2, both the total peak area and the number of effective peaks increased remarkably after optimization, with the peak area increasing by 33.66% and the peak number 15%. Results manifested that through changing extraction conditions step by step, the overall extraction effect of volatiles in pepper chicken soup could be improved obviously. Additionally, through the study of variation from peak areas of pivotal compounds, differences in extraction effect before and after optimization were further understood. ANOVA of key substances shows that the error range was mostly within 5%, indicating good repeatability of the test. Seven (1-phellandrene, linalool, ocimene, anethole, *α*-terpineol, dipropyl disulfide, and (E, E)-2,4-decadienal) of the ten key ingredients obtained various degrees of increase, among which 1-phellandrene, linalool, *α*-terpineol, and dipropyl disulfide increased significantly (p<0.05); anethole and (E, E)-2,4-decadienal) increased extremely significantly (p<0.01). Moreover, 1-phellandrene was only detected after optimization, not found before optimization. The content of sabinene had no significant change, whether optimized or not, while limonene showed an obvious decrease, which was inferred to be related to the competitive adsorption among substances during the extraction process.

## 4. Conclusion

Based on the peak area and peak number, combining variation of area of the ten key flavor substances [1-phellandrene, linalool, myrcene, limonene, ocimene, anethole, *α*-terpineol, dipropyl disulfide and (E,E)-2,4-decadienal], the SPME conditions of volatiles in pepper chicken soup were optimized by a single-factor gradual optimization method, combined with gas chromatography-mass spectrometry and principal component analysis. Firstly, according to the significant difference among the five types of fibers, the best fiber was chosen and used to optimize sample volume; then the optimal fiber and sample volume were applied in the determination of extraction temperature; finally, keeping the rest of the conditions unchanged, the three conditions chosen above were integrated to improve extraction time, and simultaneously PCA analysis conducted an effective and comprehensive evaluation. The perfect SPME conditions were identified as fiber 50/30 *μ*m DVB/CAR/PDMS, sample volume 7 g, extraction temperature 65°C, and extraction time 30 min.

With the help of advanced chromatographic methods and data algorithm, the peak area and effective peak number were both increased significantly under the conditions gained by the single-factor gradual optimization method, and, in the meantime, the key flavor compounds nearly displayed significant differences, proving the effectiveness and feasibility of the optimization pattern. Through this research, results provided important reference for the identification of aroma and the processing of pepper chicken soup and other similar products.

## Figures and Tables

**Table 1:** The five types of fibers.

Name	Coating Materials	Color	Conditioned temperature (°C)	Conditioned time (min)
85 *μ*m CAR/PDMS	Carboxen/polydimethylsiloxane	Blue	300	60
65 *μ*m PDMS/DVB	Polydimethylsiloxane/divinylbenzene	Pink	250	30
100 *μ*m PDMS	Polydimethylsiloxane	Red	250	30
50/30 *μ*m DVB/CAR/PDMS	Divinylbenzene/carboxen/polydimethylsiloxane	Gray	270	60
85 *μ*m PA	Polyacrylate	White	280	30

**Table 2:** Comparison of extraction effect before and after optimization.

Category	Peak area			Qualitative method	Significance
	Before optimization	After optimization	Change		
Total peak area	5.65E+7±7.67E+5	7.55E+7±5.38E+5	*↗*		*∗∗*
Total peak number	40.0±1.00	46.0±1.00	*↗*		*∗∗*
Sabinene	7.30E+5±4.01E+4	7.28E+5±3.31E+5	*↘*	MS/RI	
1-Phellandrene	0.00±0.00	1.09E+5±6.20E+4	*↗*	MS/RI	*∗*
Linalool	1.77E+7±2.46E+5	1.86E+7±3.96E+5	*↗*	MS/RI	*∗*
*β*-Myrcene	1.10E+6±4.59E+4	10.38E+5±2.83E+4	*↘*	MS/RI	
Limonene	7.34E+6±2.52E+5	6.44E+6±2.72E+5	*↘*	MS/RI	*∗*
Ocimene	4.88E+5±1.40E+4	5.16E+5±1.91E+5	*↗*	MS/RI	
Anethole	3.18E+6±1.53E+5	5.10E+6±1.16E+5	*↗*	MS/RI	*∗∗*
*α*-Terpineol	5.32E+5±2.36E+4	7.47E+5±1.26E+5	*↗*	MS/RI	*∗*
Dipropyl Disulfide	1.30E+5±1.07E+4	1.62E+5±1.51E+4	*↗*	MS/RI	*∗*
(E,E)-2,4-Decadienal	1.35E+5±7.40E+3	2.43E+5±8.30E+3	*↗*	MS/RI	*∗∗*

*∗* Difference is significant (p<0.05).

*∗∗* Difference is extremely significant (p<0.01).

*↗* Peak area increased after optimization.

*↘* Peak area decreased after optimization.

## Data Availability

The data used to support the findings of this study have not been made available because this involves the National Key Research and Development Program of China and our limited rights, making the data inconvenient to be opened.
